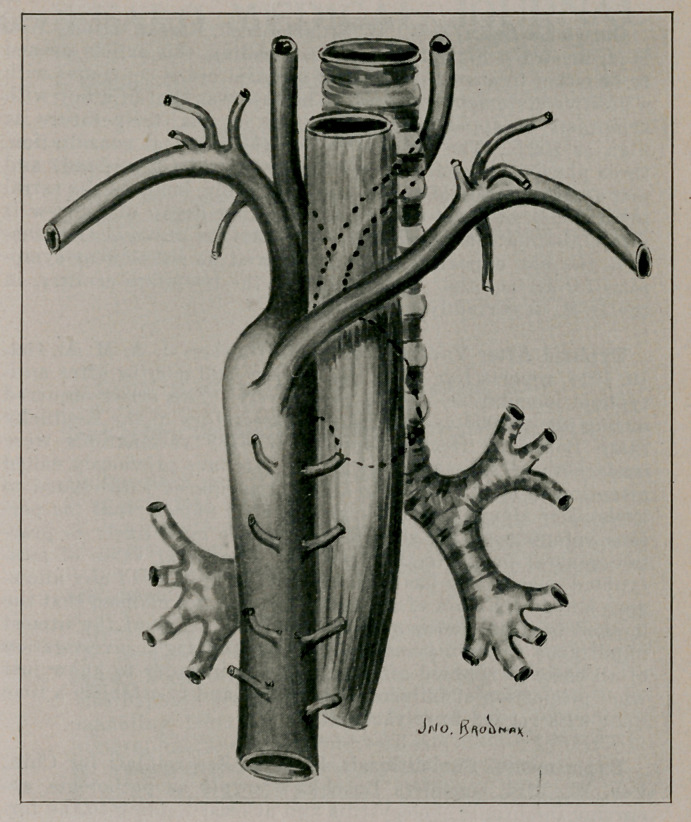# Two Cases of Abnormal Arrangement of Branches of Aortic Arch

**Published:** 1915-01

**Authors:** 


**Two Cases of Abnormal Arrangement of Branches of Aortic Arch.** Dr. John W. Broadnax, Richmond. Cuts by courtesy of Author and editor of Old Dominion Journal of Medicine and Surgery.

## Figures and Tables

**Figure f1:**
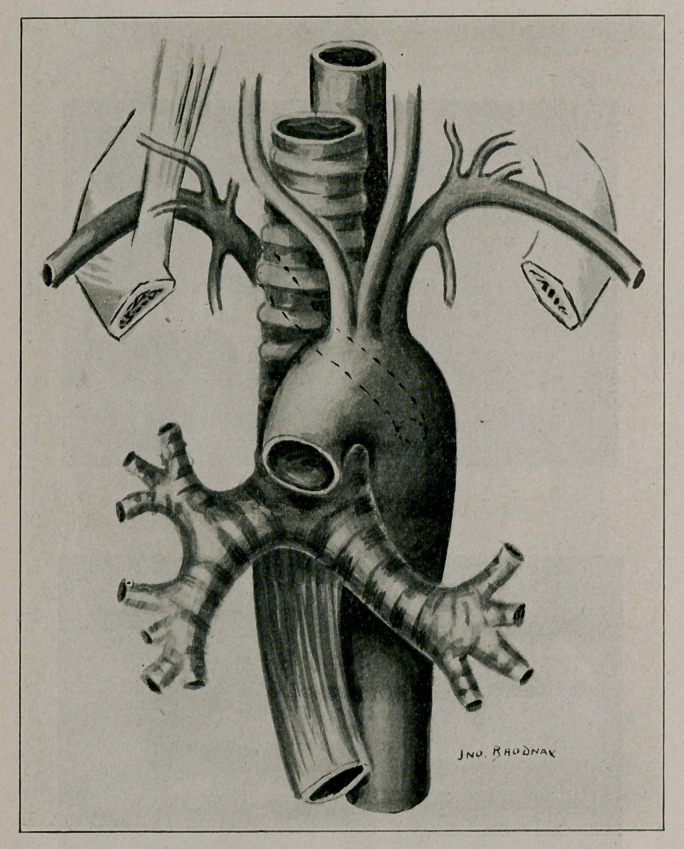


**Figure f2:**